# Fetal–neonatal exposure to antibiotics and NEC development: A systematic review and meta-analysis

**DOI:** 10.3389/fped.2022.1102884

**Published:** 2023-01-16

**Authors:** Daphne H. Klerk, Lisanne K. van Avezaath, Erik A. H. Loeffen, Jan B. F. Hulscher, Elisabeth M. W. Kooi

**Affiliations:** ^1^ Division of Neonatology, Beatrix Children's Hospital, University of Groningen, University Medical Center Groningen, Groningen, Netherlands; ^2^ Division of Pediatric Oncology/Hematology, Beatrix Children's Hospital, University of Groningen, University Medical Center Groningen, Groningen, Netherlands; ^3^ Division of Pediatric Surgery, Department of Surgery, University of Groningen, University Medical Center Groningen, Groningen, Netherlands

**Keywords:** necrotizing enterocolitis (NEC), empirical antibiotic, prolonged antibiotic therapy, maternal antibiotics, systematic review, meta-analysis, preterm, neonate

## Abstract

**Background:**

Fetal and neonatal exposure to antibiotics may contribute to the development of necrotizing enterocolitis (NEC) in preterm infants. This systematic review and meta-analysis investigate whether exposure to third trimester maternal antibiotics (MAB) and/or prolongation of empirical antibiotics (PEAB) are associated with NEC development in preterms.

**Method:**

We included observational and randomized controlled studies, including those on preterm or very low birth weight (VLBW) infants, from MEDLINE and EMBASE, published between 1990 and June 2021. Exposure was defined as third trimester MAB and/or PEAB. The two reviewers independently performed study selection, data extraction, and quality assessment.

**Results:**

Three cohort studies compared third trimester MAB with no antibiotics. MAB was associated with lower NEC incidence, unadjusted pooled odds ratio (OR) is 0.57 (95% CI: 0.35–0.93). Twelve cohort studies showed that PEAB was associated with an increased risk of NEC. Ten observational cohort studies show an unadjusted OR of 2.72 (1.65–4.47), and two case–control studies show an unadjusted mean difference of 2.31 (0.94–3.68). Moderate to substantial heterogeneity was observed but decreased in studies with low risk of bias and large sample size.

**Conclusion:**

Evidence suggests an association between MAB and decreased risk of NEC and an association between PEAB and increased risk of NEC. Further studies should confirm these associations and explore causality.

**Systematic Review Registration:**

identifier [CRD42022304937].

## 1. Introduction

Necrotizing enterocolitis (NEC) is an inflammatory bowel disease and the most common gastrointestinal emergency in newborn infants. Reaching its peak incidence around 31 weeks of postconceptional age, NEC primarily affects preterm-born neonates (1, 2). NEC may progress within hours from subtle symptoms to a critical condition and can eventually result in death (3). The rate of death associated with NEC is 15%–30%, and it is one of the leading causes of morbidity in the NICU (1, 4). In surviving neonates, intestinal strictures and short bowel syndrome can be observed, as well as a risk of neurodevelopmental impairment (5, 6).

One of the known risk factors for NEC development is alterations in the intestinal microbiota colonization of the neonate. Differences in the microbiota of infants who develop NEC can already be found in the meconium (7). Generally, an increase in gram-negative bacteria and a decrease in anaerobic bacteria are associated with NEC, suggesting a microbial dysbiosis (8). The microbiota can be influenced by multiple factors, both during and after pregnancy, such as the maternal diet, type of feeding, gestational age, delivery type, length of hospitalization, and infections (9, 10). In addition, the neonatal microbiota is influenced by exposure to antibiotics, both during and after pregnancy. Antibiotic exposure early in life can stall the development of the intestinal microbiota in the developing gut, resulting in a decrease in microbial diversity (11). Preterm-born infants are exposed to antibiotics *in utero* in cases of maternal antibiotic use. Mothers are administered antibiotics in cases of preterm premature rupture of membranes (PPROM), chorioamnionitis, as group B streptococcus (GBS) prophylaxis, prior to a cesarean section and for suspected intrauterine infections. Microbiota colonization of the neonate is suspected to have already started at this time (7, 9, 12–14). Maternal antibiotic use is associated with a significant decrease in alpha diversity, even if only used intrapartum (15).

Neonatal empirical antibiotics (EAB) are initiated immediately after preterm birth, for neonates who are deemed at risk for developing early onset neonatal sepsis (16). This empiric antibiotic use is often ceased after a negative blood culture. However, empiric antibiotics are prolonged in up to 29% of neonates for longer than 48 h, despite a negative blood culture (17). A short course of antibiotics after birth only affects infant microbiota diversity temporarily, whereas longer term antibiotics (>3 or 4 days) result in a sustained reduction in microbiota diversity (18, 19).

Exposure to antibiotics in preterm-born infants, in pregnancy or shortly after birth may contribute to the development of NEC. Given the dramatic consequences for the newborn, identifying all risk factors that can contribute to the development of NEC, is highly important. The purpose of this systematic review and meta-analysis is to assess the risk of developing NEC in infants that were exposed to antibiotics in the third trimester, compared with infants that were not exposed to maternal antibiotic use (MAB). We will also compare the risk of NEC development in infants receiving prolonged empiric antibiotics (PEAB) with infants receiving nonprolonged EAB. As a secondary outcome, we will assess the association between maternal and neonatal antibiotics and infant mortality. This is the first review to explore the full exposure to antibiotics of the neonate, both *in utero* through maternal antibiotic use as well as after birth.

## 2. Methods

This protocol was registered within the International Prospective Register of Systematic Reviews (PROSPERO) database (registration ID CRD42022304937). Following the completion of the literature research, this study was reviewed and accepted into the register; however, the protocol adheres to the Preferred Reporting Items for Systematic Reviews and Meta-Analyses (PRISMA) statement (Supplementary File S1) (20).

### 2.1. In/exclusion criteria

Studies were selected according to the following criteria:

#### 2.1.1. Studies

Articles must include human subjects. Randomized controlled trials (RCTs), retrospective and prospective cohorts, and case–control studies published between 1990 and 20th June 2021 were included. English-written and translated studies that were originally written in a language other than English were included. Case reports, systematic reviews, and meta-analyses, as well as studies based on animal research, were excluded.

#### 2.1.2. Participants

Preterm-born infants, born with a gestation of 32 weeks or less, very low birth weight infants with a birth weight of ≤1,500 g, or extremely low birth weight neonates (≤1,000 g), with NEC and controls, were included.

#### 2.1.3. Interventions and comparisons

For maternal antibiotic exposure, administration of antibiotics during the last trimester of pregnancy, was compared with no administration of prenatal antibiotics. For neonatal antibiotic exposure, administration of EAB was compared with prolonged administration (PEAB) according to the author's definition. Studies on maternal antibiotic use in which no control group was used were excluded. Additionally, studies in which empiric neonatal antibiotic administration was solely compared with no neonatal antibiotic use were also excluded, as this was not part of the research question.

#### 2.1.4. Outcomes

The primary outcome was the prevalence of confirmed NEC (Bell's stage 2 or higher). As a secondary outcome, the infant mortality rate was assessed as defined by the authors.

### 2.2. Search methods for identification of studies

Relevant studies were identified through systematic searches within the MEDLINE and EMBASE databases. Other potentially eligible studies were identified by backward reference searching of systematic reviews and meta-analyses, as well as the Cochrane database of reviews. The detailed search strategy for MEDLINE, EMBASE, and Cochrane can be found in Supplementary File S2. No limits or filters were used. Screening based on publication year and language was done manually.

### 2.3. Data collection and analysis

**Figure 1:**
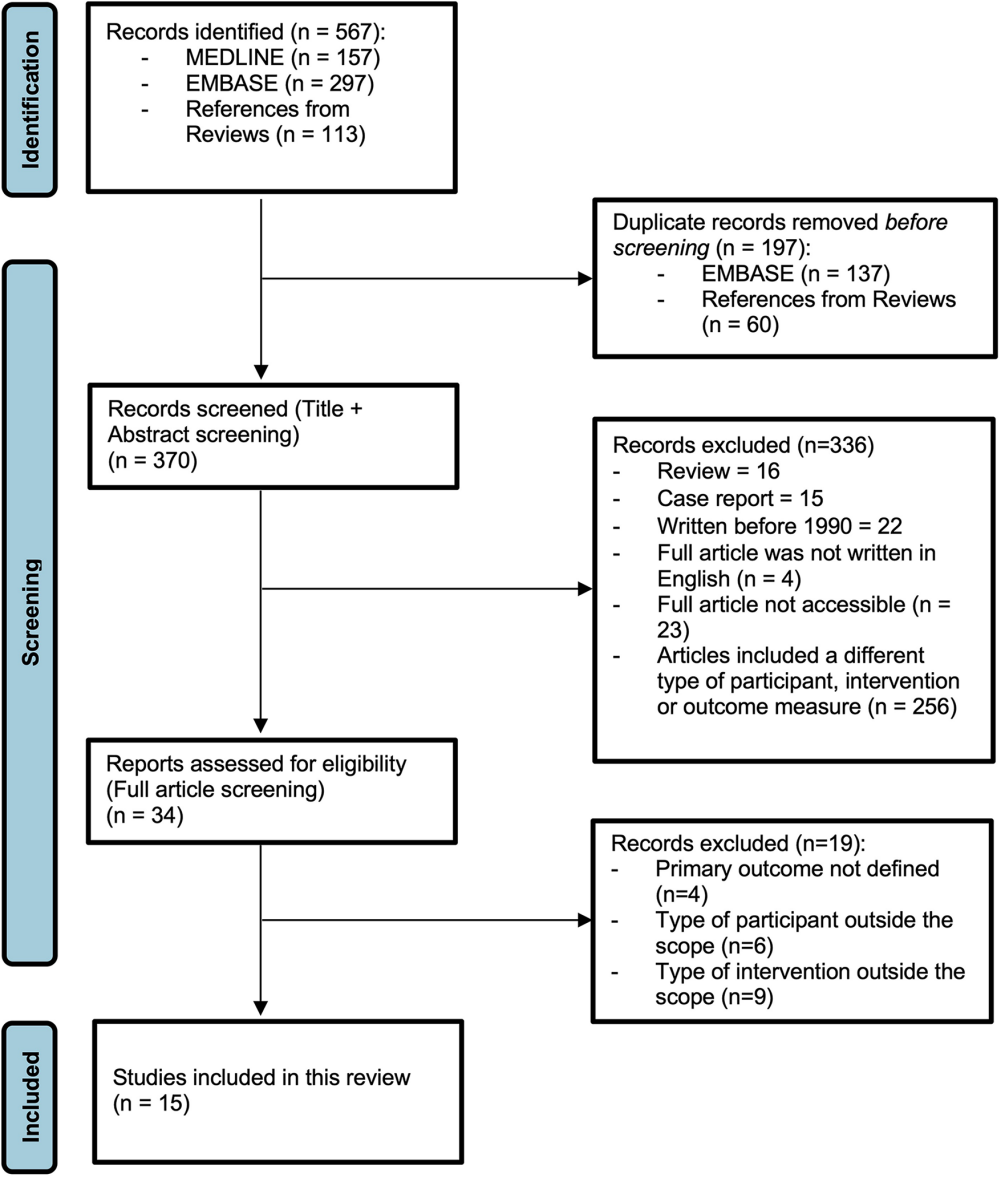
PRISMA flow diagram (19).

Studies were retrieved from the search strategy, after which they were screened by title and abstract, and included or excluded after full text screening by two independent researchers (DHK and LKvA). Databases were screened between 15 April 2021 and 20 June 2021. The process of article screening for inclusion and exclusion criteria is presented in a flow diagram (Figure 1). From the included studies, data were extracted independently by two researchers (DHK and LKvA). Any inconsistencies between the two researchers were discussed with a third independent researcher (EMWK). For all included articles, the extracted data were presented in characteristic tables (Tables 1–4). No automation tools were used for this process.

**Table 1:** Characteristics of studies on maternal antibiotics use.

Study— maternal AB	Country	Patients	Infants with NEC	Method	MAB	No MAB	Type of AB	Infant mortality
Boo, 2012	Malaysia	*N*=3,601 (≤1,500 g)	*N*=222 (6.2%)	RCS	AB in 24 h prior to delivery	No AB in 24 h prior to delivery	Unknown	Death before transfer or discharge
Mercer, 1997	United States	*N*=495 (≤32 weeks)	*N*=29 (4.1%)	RCT	7-day AB treatment after PPROM	No AB after PPROM	Ampicillin, erythromycin, and amoxicillin	Death before transfer or discharge
Reed, 2018	United States	*N*=580 (<32 weeks)	*N*=44 (7.6%)	PCS	AB in 72 h prior to delivery	No AB in 72 h prior to delivery	Cefazolin, ampicillin, penicillin G, vancomycin, clindamycin, ampicillin+azithromycin	Death between birth and 120 postnatal days or discharge
Boo, 2012	Malaysia	*N*=3,601 (≤1,500 g)	*N*=222 (6.2%)	RCS	AB in 24 h prior to delivery	No AB in 24 h prior to delivery	Unknown	Death before transfer or discharge
Mercer, 1997	United States	*N*=495 (≤32 weeks)	*N*=29 (4.1%)	RCT	7-day AB treatment after PPROM	No AB after PPROM	Ampicillin, erythromycin, and amoxicillin.	Death before transfer or discharge
Reed, 2018	United States	*N*=580 (<32 weeks)	*N*=44 (7.6%)	PCS	AB in 72 h prior to delivery	No AB in 72 h prior to delivery	Cefazolin, ampicillin, penicillin G, vancomycin, clindamycin, ampicillin+azithromycin	Death between birth and 120 postnatal days or discharge

MAB, maternal antibiotics; AB, antibiotics; NEC, necrotizing enterocolitis; RCS, retrospective cohort study; PCS, prospective cohort study; RCT, randomized controlled trial; PPROM, preterm premature rupture of membranes.

**Table 2:** Maternal antibiotics use compared with no maternal antibiotics use.

Study—maternal AB	No MAB group	MAB group	NEC no MAB group	NEC MAB group	CS no MAB group	CS MAB group	Odds ratio (95% CI)	*p* value	Infant mortality no MAB group	Infant mortality MAB group	Odds ratio (95% CI)	*p* value
Boo, 2012	2,783 (77.3%)	818 (22.7%)	180 (6.5%)	42 (5.1%)	Unknown	Unknown	0.64 (0.42–0.97)^a^	0.04	Unknown	Unknown	Unknown	Unknown
Mercer, 1997	257 (51.9%)	238 (48.0%)	14 (5.4%)	5 (2.1%)	80 (31.1%)	70 (29.4%)	0.37 (0.13, 1.05)^b^	0.06^b^	15 (5.8%)	13 (5.5%)	0.51 (0.26, 1.02)^b^	0.06^b^
Reed, 2018	218 (37.6%)	362 (62.4%)	25 (11.5%)	19 (5.2%)	167 (76.6%)	96 (54.1%)	0.28 (0.14–0.56)^a^	<0.001	26 (11.9%)	30 (8.3%)	0.29 (0.14–0.60)^a^	<0.001

MAB, maternal antibiotics use; NEC, necrotizing enterocolitis; CS, cesarean section; CI, confidence interval.

^a^ Adjusted odds ratios.

^b^ Odds ratios in this table are author reported; in absence thereof, RevMan calculated odds ratios were added.

**Table 3:** Characteristics of studies on prolonged empirical antibiotics use in the neonate.

Study—neonatal EAB	Country	Patients	Infants with NEC	Method	EAB	PEAB	Type of AB used	Mortality
Abdel Ghany, 2012	Egypt	*N* = 207 (≤1,500 g)	*N* = 34 (16.4%)	RCS	1–4 days	≥5 days	Ampicillin, gentamicin	Unknown
Afjeh, 2016	Iran	*N* = 145 (≤1,500 g)	*N* = 5 (3.4%)	PCS	≤2 weeks	>2 weeks	Ampicillin, gentamicin, vancomycin, cefotaxim, amikacin, ceftazidime, meropenem, gentamicin, vancomycin, cefotaxim, amikacin, ceftazidim, meropenem	Death after 12 days of life
Al-Mouqdad, 2018	Saudi Arabia	*N* = 295 (≤32 weeks or <1,500 g)	*N* = 102 (34.6%)	RCS	1–5 days	≥6 days	Unknown	Death before transfer or discharge
Alturk, 2021	Qatar	*N* = 199 (<29 weeks)	*N* = 35 (17.6%)	RCS	<48 h	>48 h	Cefotaxim, ampicillin, amikacin, penicillin	Death before transfer or discharge
Cotten, 2009	United States	*N* = 4,039 (<1,000 g)	*N* = 440 (10.9%)	RCS	<5 days	≥5 days	Ampicillin, gentamicin, cefotaxim, other	Death between birth and 120 postnatal days or discharge
Esmaeilizand, 2018	Canada	*N* = 671 (<29 weeks)	*N* = 224 (33.4%)	RCCS	7 ± 6 days	10 ± 8 days	Unknown	Unknown
Fajardo, 2019	Canada	*N* = 620 (<1,250 g)	*N* = 18 (2.9%)	RCS	≤5 days	>5 days	Unknown	Death after 5 days of life
Kuppala, 2011	United States	*N* = 365 (≤32 weeks or ≤1,500 g)	*N* = 17 (4.7%)	RCS	1–4 days	≥5 days	Ampicillin, gentamicin, clindamycin, amphoterecin B, nafcillin, cefotaxime, erythromycin	Death between birth and 120 postnatal days or discharge
McGrath, 2011	Ireland	*N* = 98 (<1,000 g)	*N* = 22 (22.4%)	RCS	<3 days	≥3 days	Unknown	Unknown
Raba, 2019	Ireland	*N* = 54 (<1,500 g)	*N* = 21 (40.4%)	RCCS	2.7 ± 2.3 days	4.3 ± 2.1 days	Amoxicillin, gentamicin, benzyl penicillin, cefotaxim, vancomycin, meropenem	Unknown
Ting, 2019	Canada	*N* = 14,207 (<1,500 g)	N = 531 (3.7%)	RCS	1–3 days	4–7 days	Unknown	Death after 7 days of life
Torres, 2018	Chile	*N* = 213 (<32 weeks or <1,500 g)	*N* = 20 (9.4%)	CS	1–4 days	≥5 days	Ampicillin, amikacin	Unknown

EAB, empirical antibiotics use; PEAB, prolonged EAB; NEC, necrotizing enterocolitis; RCS, retrospective cohort study; PCS, prospective cohort study; RCCS, retrospective case–control study; CS, cross-sectional study; AB, antibodies.

**Table 4:** Empirical antibiotics use compared with prolonged empirical antibiotics use.

Study—neonatal EAB	EAB	PEAB	NEC in EAB group	NEC in PEAB group	Odds ratios (95% CI)	*p* value	Mortality EAB group	Mortality PEAB group	Odds ratios (95% CI)	*p* value
Abdel Ghany, 2012	*n* = 34 (16.4%)	*n* = 173 (83.6%)	0 (0%)	34 (19.7%)	1.31 (1.04–1.65)	0.18	0 (0%)	80 (46.2%)	1.44 (1.23–1.68)	<0.001
Afjeh, 2016	*n* = 62 (42.8%)	*n* = 83 (57.2%)	1 (1.6%)	4 (4.8%)	3.09 (0.34–28.34)^a^	0.32	0 (0%)	12 (14.5%)	0.12 (0.06, 0.27)^b^	<0.001^b^
Al-Mouqdad, 2018	*n* = 104 (35.3%)	*n* = 191 (64.7%)	5 (4.8%)	97 (50.8%)	20.43 (7.96–52.42)^b^	<0.001^b^	36 (34.6%)	28 (14.7%)	36.43 (2.20, 603.17)^b^	0.01^b^
Alturk, 2021	*n* = 14 (7%)	*n* = 186 (93%)	1 (7.1%)	34 (18.3%)	2.91 (0.37–22.99)^b^	0.31^b^	1 (7.1%)	14 (7.5%)	1.06 (0.13, 8.69)^b^	0.96^b^
Cotten, 2009	*n* = 1,892 (46.8%)	*n* = 2,147 (53.2%)	185 (9.8%)	255 (11.9%)	1.21 (0.98–1.51)^a^	0.08	245 (12.9%)	412 (19.2%)	1.46 (1.19–1.78)^a^	<0.001
Esmaeilizand, 2018	*n* = 447	*n* = 224	7 ± 6 days	10 days ± 8	2.02 (1.55–3.13)^a^	Unknown	Unknown	Unknown	Unknown	Unknown
Fajardo, 2019	*n* = 382 (61.6%)	*n* = 238 (38.4%)	7 (1.8%)	11 (4.6%)	1.58 (0.54–4.61)^a^	Unknown	10 (2.6%)	9 (3.8%)	1.46 (0.59, 3.65)^b^	0.42^b^
Kuppala, 2011	*n* = 175 (47.9%)	*n* = 130 (35.6%)	8 (4.6%)	9 (6.9%)	1.28 (0.42–3.93)^a^	0.66	8 (4.6%)	12 (9.2%)	1.12 (0.40–3.10)^a^	0.83
McGrath, 2011	*n* = 14 (14.3%)	*n* = 84 (85.7%)	1 (7%)	21 (25%)	4.3 (0.6–192.8)	0.18	Unknown	Unknown	Unknown	Unknown
Raba, 2019	*n* = 31	*n* = 21	2.7 ± 2.3 days	4.3 days ± 2.1	3.6 (1.13–11.47)	0.05	Unknown	Unknown	Unknown	Unknown
Ting, 2019	*n* = 5,401 (38%)	*n* = 5,856 (41.2%)	177 (3.3%)	273 (4.7%)	0.87 (0.55–1.39)^a^	Unknown	144 (2.7%)	354 (6%)	0.74 (0.47–1.17)^a^	Unknown
Torres, 2018	*n* = 69 (32.4%)	*n* = 144 (67.6%)	0 (0%)	20 (13.9%)	9.71 (1.27–74.35)	0.03	Unknown	Unknown	3.35 (0.73–15.28)	0.12

EAB, empirical antibiotics use; PEAB, prolonged EAB; NEC, necrotizing enterocolitis; CI, confidence interval.

For the two case control studies, the column EAB group contains the mean number of days of antibiotics given in the control group, with the SD. The PEAB group contains the mean number of days of antibiotics given in the cases, the NEC infants, before NEC onset.

^a^ Odds ratios and p values in this table are author reported, in absence thereof RevMan calculated odds ratios were added.

^b^ Adjusted odds ratios.

### 2.4. Assessment of the risk of bias in included studies

The risk of bias was assessed by two independent reviewers (DHK and LKvA) for all included studies using the Cochrane risk-of-bias tool for randomized trials (RoB 2) or the Risk Of Bias In Non-randomized Studies—of Interventions (ROBINS-I) tool for observational studies (21, 22).

### 2.5. Measures of associations

The data were reported in absolute numbers, percentages, and odds ratios (OR) with 95% conﬁdence intervals (CIs). Due to the difference in study design, case–control studies were analyzed separately from observational cohort studies and randomized controlled trials. For case–control studies, NEC cases were compared with controls, and the mean differences (MD) in days of antibiotic treatment, as well as the CIs, between both groups were reported. In addition, we performed a subgroup analysis to pool clinically similar studies and investigate if a similar intervention effect was present for subgroups (Table 5). This included studies where maternal antibiotics were given shortly before birth, and a subgroup including extremely preterm-born infants born with a gestation of <30 weeks or a birth weight <1,000 g. Finally, as a sustained reduction in microbiota diversity is seen after 3 or 4 days of antibiotics, and current standards suggest evaluating the necessity of antibiotics at 36–48 h, we evaluated the subgroup receiving antibiotics for ≤3 days in the control group compared with prolonged antibiotic treatment >3 days. If necessary, the required data were calculated using the data presented in the studies.

**Table 5:** Sensitivity and subgroup analysis of all included observational cohort studies comparing EAB and PEAB.

	Study (*n*)	Random-effects OR (95% CI)	*p* value	*I* ^2^	*p* value
Sample size					
>200	7	2.66 (1.54–4.59)	<0.001	86%	<0.001
>500	3	1.37 (1.14–1.65)	<0.001	30%	0.24
>1,000	2	1.34 (1.16–1.55)	<0.001	9%	0.29
Risk of bias					
Low	2	5.73 (0.92–35.59)	0.06	14%	0.28
Subgroups					
Birth weight <1,000 g or gestational age <30 weeks	3	1.27 (1.04–1.55)	0.02	0%	0.37
≤3 days of EAB	3	1.47 (1.21–1.77)	<0.001	0%	0.48

EAB, empirical antibiotics; PEAB, prolonged empirical antibiotics; CI, confidence interval; OR, odds ratio.

### 2.6. Missing data

Reasons for the missing data were investigated, and when missing data were thought critical for this review, authors were contacted. Studies were regarded as having a high risk of bias for incomplete outcome data if 20% or more outcome data were missing, or if missing data were not reported.

### 2.7. Assessment of heterogeneity

Heterogeneity was assessed by visual inspection of the forest plot and by using the *I*^2^ statistic. In cases of substantial heterogeneity (*I*^2^ > 50%) (23), potential causes were investigated through sensitivity analyses, including only studies with a low risk of bias and studies with a larger sample size. The pooled odds ratio of these subgroups was compared with the original pooled OR to evaluate if a similar intervention effect was present.

### 2.8. Assessment of reporting biases

To assess publication bias, we made a funnel plot in the case of the inclusion of 10 or more studies (24). In cases of asymmetry on visual inspection, results should be interpreted with caution. In addition, in cases of significant heterogeneity and asymmetry in the funnel plot, we presented both the random and fixed effects estimates of the intervention effect to evaluate if a similar intervention effect was present in both models.

### 2.9. Data synthesis

Meta-analytic software [*Review Manager (RevMan) Computer program. Version 5.4, The Cochrane Collaboration, 2020*] was used for this review, and the OR was calculated using a random-effects model. Unadjusted self-calculated odds ratios were used for the meta-analysis, since primary data meta-analysis was not possible at this moment. Case–control studies were evaluated separately due to the differences in study design.

### 2.10. Quality of evidence

The quality of evidence for the primary outcome, NEC, was graded using the Grading of Recommendations, Assessment, Development, and Evaluations (GRADE).

## 3. Results

### 3.1. Search results

The MEDLINE search yielded 157 records, the EMBASE search yielded 297 records, and references in reviews from the Cochrane Library yielded 113 records. A total of 197 duplicate records were excluded, leaving 370 records to be screened based on title and abstract (Figure 1).

### 3.2. Excluded studies

After the title and abstract screenings, 34 studies were screened in full. Of these, four articles did not use a clear NEC definition based on Bell's criteria (25–27). All first authors were contacted, only one replied, indicating that indeed no formal criteria for NEC diagnosis were used (26). As a consequence, these four studies were excluded. Six articles included infants older than 32 weeks of gestation and did not mention infants born very low birth weight (VLBW) or ≤32 weeks as a subgroup in their analyses (28–34). Another five articles did not include a clear comparison between EAB use and PEAB for infants with NEC. The first by Greenwood et al. used a compound outcome instead of a NEC definition (18). Alsafadi et al. did not specify the distribution of NEC infants among both groups (35). Lewis et al. and Segel et al. did not include a control group for maternal antibiotic use (36, 37). In the article by Hosseini et al. the antibiotics in the control group were stopped after CRP was negative (38). Finally, four articles compared AB use with no AB use, instead of empiric vs. prolonged use (39–42).

### 3.3. Included studies

The inclusion criteria were met by 15 articles: 3 articles compared MAB use in the third trimester with no antibiotics use, and 12 articles were included for comparing PEAB vs. EAB use (17, 43–56). Nine retrospective cohort studies, two prospective cohort studies, two case–control studies, one cross-sectional observational study, and one randomized controlled trial were included. There was no study was found that evaluated both MAB use in the third trimester and PEAB use in preterm infants. The characteristics and outcomes of all included studies are presented in Tables 1–4.

#### 3.3.1. Participants

A total of 25,788 patients were included, divided in 4,676 mothers and 21,112 infants (Tables 1, 3).

#### 3.3.2. Intervention

Administration of antibiotics during the last trimester of pregnancy was compared with no administration of prenatal antibiotics. Two studies included antibiotic use shortly before delivery, and one study included antibiotic use for 7 days after PPROM (43–45). For neonatal antibiotic exposure, administration of empirical antibiotics was compared with prolonged administration, ranging from 3 days to 2 weeks.

#### 3.3.3. Outcomes

All studies included the incidence of NEC, defined as Bell's stage 2 or higher, in the intervention and control groups. In addition, several articles reported overall mortality. The definition of mortality varied, e.g. after a set number of days of life or before discharge. See Tables 1, 3 for details.

### 3.4. Risk of bias

Fourteen observational studies were assessed using the ROBINS-I tool, and one RCT was assessed using the ROB2 tool. The results can be found in Table 6. The most important finding was the presence of bias due to confounding in the majority of included studies, which can be explained by the presence of many observational cohort studies in this meta-analysis.

**Table 6:** Risk of bias score for all included studies.

Article	ROBINS-1							ROB2				
	1	2	3	4	5	6	7	A	B	C	D	E
Maternal												
Boo, 2012	+	−	−	−	/	−	−					
Mercer, 1997								−	−	+	−	−
Reed, 2018	+	−	−	−	/	−	−					
Neonatal												
Abdel Ghany, 2012	+	−	−	−	/	−	−					
Afjeh, 2016	+	−	−	−	/	−	−					
Al-Mouqdad, 2018	+	−	−	−	−	−	−					
Alturk, 2021	−	−	−	−	/	−	−					
Cotten, 2009	+	−	−	−	−	−	−					
Esmaeilizand, 2018	−	+	−	−	/	−	−					
Fajardo, 2019	+	−	−	−	/	−	−					
Kuppala, 2011	+	−	−	−	−	−	−					
McGrath, 2011	+	/	−	−	/	−	−					
Raba, 2019	−	−	−	−	+	−	−					
Ting, 2019	+	−	−	−	+	−	−					
Torres, 2018	−	−	−	−	/	−	−					

ROBINS-I tool: (1) Bias due to confounding, (2) Selection of participants into the study, (3) Bias in classification of the interventions, (4) Bias due to deviation from intended interventions, (5) Bias due to missing data, (6) Bias in measurements of outcome, and (7) Bias in selection of the reported result. ROB2 tool: (A) Bias arising from the randomization process, (B) Bias due to deviations from intended interventions, (C) Bias due to missing outcome data, (D) Bias in measurement of the outcome, and (E) Bias in selection of the reported result. A plus sign indicates a high risk of bias, whereas a minus sign indicates a low risk of bias, and a slash indicates that this category could not be scored due to insufficient information.

### 3.5. Assessments of certainty in the body of evidence

The certainty of the evidence was assessed using GRADE for the primary outcome of NEC. Since most of the included studies were observational, the starting level of evidence was low. Reasons for downgrading were a substantial risk of bias and the probability of publication bias (Figure 5). The dose–response effect reported in several studies was the reason for upgrading. This results in a very low-quality overall body of evidence for the primary outcome of NEC.

### 3.6. Measures of associations

#### 3.6.1. NEC

**Figure 2:**
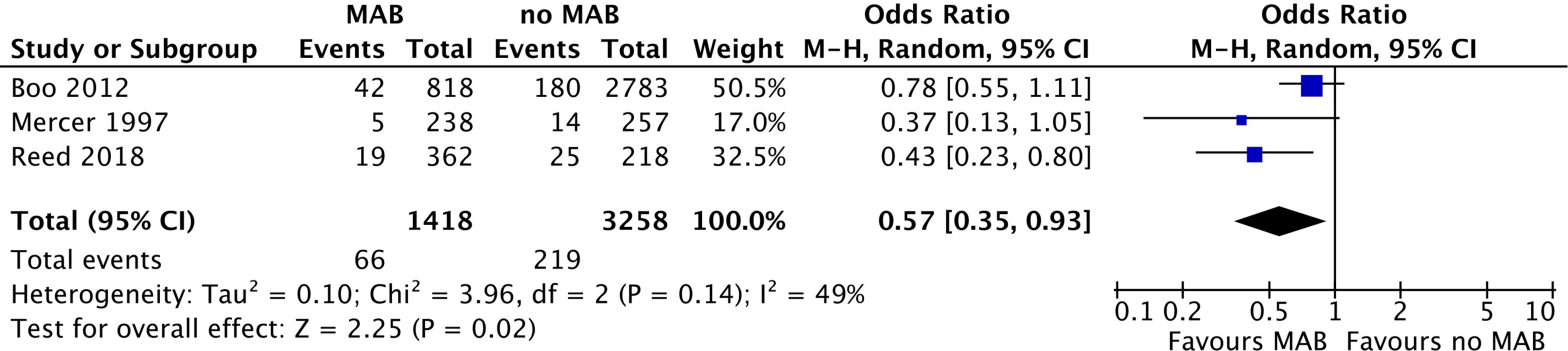
Forest plot of all included studies comparing MAB and no MAB. Events=the incidence of NEC development. Odds ratios were calculated using RevMan. MAB, maternal antibiotics; NEC, necrotizing enterocolitis; CI, confidence interval.

Two studies investigating MAB reported a significantly lower incidence of NEC in the groups receiving MAB compared with the group that did not, one study showed no difference. The pooled odds ratio for NEC by the random effect model is 0.57 (95% CI: 0.35–0.93, *p*=0.02) for infants receiving MAB compared with infants receiving no MAB (Figure 2). The subgroup including the two studies where MAB was given shortly before birth showed a pooled odds ratio for NEC by the random effect model is 0.61 (95% CI: 0.34–1.10, *p*=0.10).

**Figure 3:**
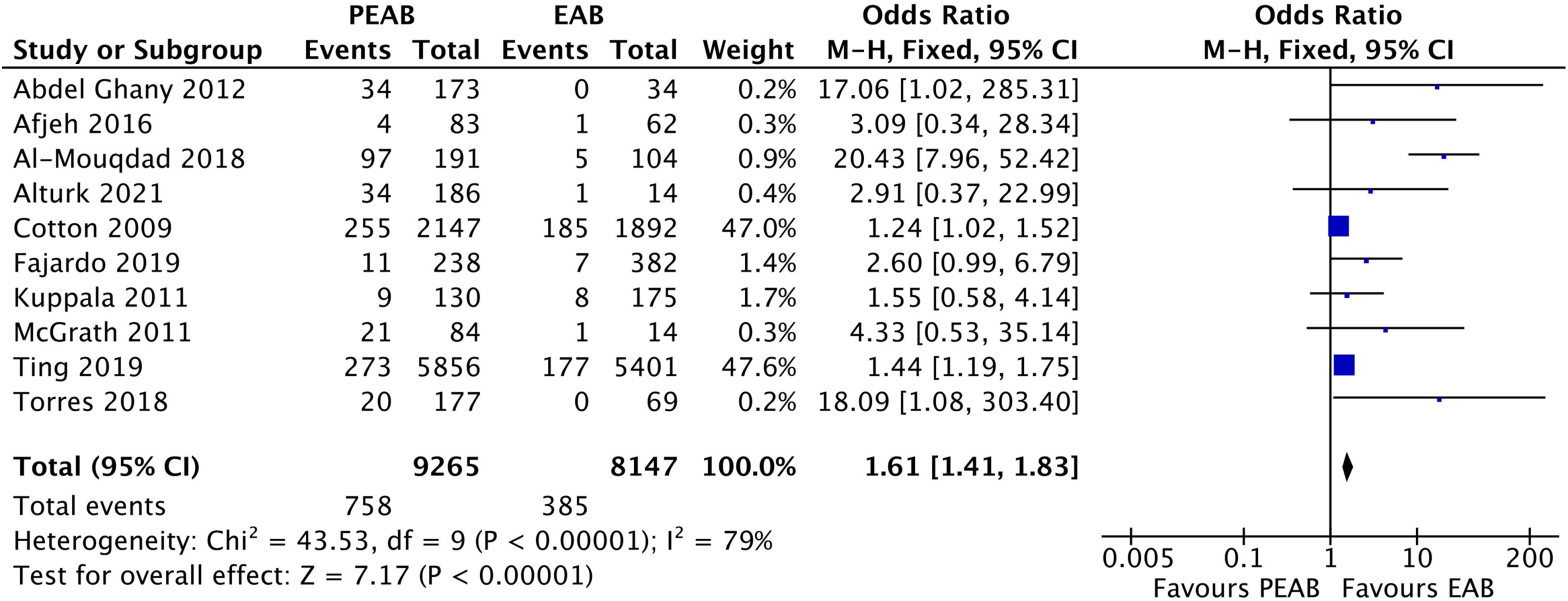
Forest plot of all included observational cohort studies comparing EAB and PEAB. Events=the incidence of NEC development. Odds ratios were calculated using RevMan, EAB, empirical antibiotics use; PEAB, Prolonged EAB; NEC, necrotizing enterocolitis; CI, confidence interval.

From 10 observational cohort studies comparing the prevalence of NEC in infants receiving PEAB with EAB, 5 reported a significantly higher prevalence of NEC in the PEAB group (Table 4). The pooled odds ratio for NEC was 2.72 (95% CI: 1.65–4.47, *p*<0.0001) for infants receiving PEAB compared with infants receiving EAB (Figure 3). In the individual studies that corrected for confounders, no significant odds ratios remained. The increased prevalence of NEC after prolongation of empirical antibiotics ranged between 2% and 46% compared with the control group. Only the studies by Cotten et al. and Ting et al. reporting adjusted odds ratios, did not show that the incidence of NEC after prolonging empirical antibiotics at least doubled. Finally, we found pooled odds ratios indicating an increased risk of NEC in the PEAB groups, including only very preterm infants (<30 weeks of gestation or born with a birth weight <1,000 g) and infants in the EAB group receiving only ≤3 days of antibiotics in the EAB group (Table 5).

**Figure 4:**
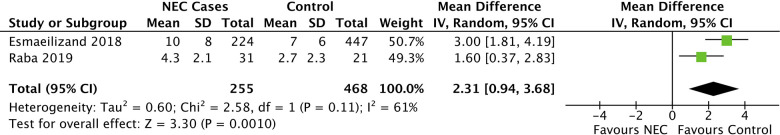
Forest plot of case–control studies comparing EAB and PEAB. For NEC cases, antibiotic treatment in days before NEC onset was compared with controls. Mean differences were calculated using RevMan. EAB, empirical antibiotics use; PEAB, Prolonged EAB; NEC, necrotizing enterocolitis; CI, confidence interval.

Infants with NEC received empiric antibiotics for more days before the onset of NEC in the case–control studies of Esmaeilizand et al. and Raba et al. compared with infants who did not develop NEC (Table 4). These differences were significant in both studies. The pooled mean difference was 2.31 days (95% CI: 0.94–3.68, *p*=0.001), for infants in the control group compared with the NEC cases (Figure 4).

#### 3.6.2. Heterogeneity

The studies comparing MAB with no MAB showed moderate heterogeneity (*I*^2^=49%, *p* value=0.14) and were not further evaluated. There was substantial heterogeneity in the observational cohort studies evaluating PEAB vs. EAB (*I*^2^=79%, *p* value<0.001). Heterogeneity that was present in the overall meta-analysis was partially explained in the sensitivity analysis with stratification by sample size and risk of bias. The heterogeneity was low or negligible in the subgroups with a sample size of >500 infants and >1,000 infants and in studies with a low risk of bias, while the pooled effect size remained statistically significant (Table 5). There was also substantial heterogeneity in the meta-analysis performed for the two case–control studies (*I*^2^=61, *p* value=0.09). This could not be further analyzed using subgroups, due to the presence of only two groups in this analysis.

#### 3.6.3. Assessment of publication bias

**Figure 5:**
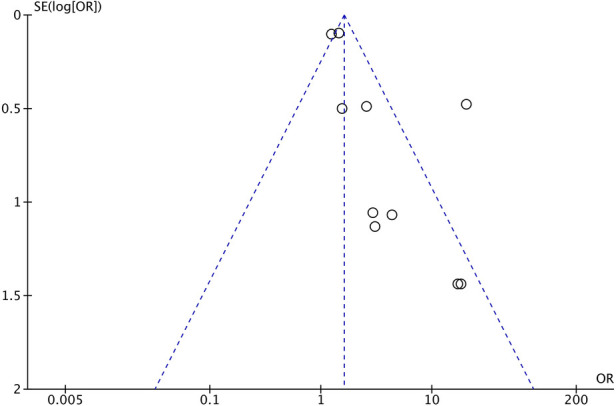
Funnel plot for observational cohort studies comparing EAB and PEAB. Odds ratios were calculated using RevMan. EAB, empirical antibiotics use; PEAB, prolonged EAB; NEC, necrotizing enterocolitis; SE, standard error; OR, odds ratio.

We included 10 observational cohort studies in the meta-analysis and constructed a funnel plot for these ten studies (Figure 5). This funnel plot shows asymmetry on visual inspection. One outlier can be seen in the study by Al-Mouqdad et al. Due to the presence of substantial heterogeneity, we did not perform any tests for funnel plot asymmetry (e.g., Egger's test). The test power is too low to distinguish chance from real asymmetry, and the minimum number of studies may be substantially more than 10 in this case (24). The fixed effects model for all observational studies comparing PEAB with EAB showed a significant pooled odds ratio of 1.61 (95% CI: 1.41–1.83, *p*<0.001). When excluding the study from Al-Mouqdad et al. the random-effects model for all observational studies comparing PEAB with EAB showed a significant pooled odds ratio of 1.53 (95% CI: 1.18–1.97, *p*=0.001) with an *I*^2^ of 31%.

#### 3.6.4. Mortality

Two out of three studies comparing MAB with no MAB report no significant differences in mortality rates between the two groups (Table 2) (44, 45). Three studies report a significantly lower mortality in the EAB group, compared with four that do not report this as a significant finding (Table 4). The only study showing a decrease of 19% in mortality after prolonging EAB was the study performed by Al-Mouqdad et al. (48).

## 4. Discussion

This meta-analysis, based on 15 studies evaluating the full neonatal exposure to antibiotics, shows that MAB was associated with a reduced risk of NEC development, whereas PEAB use was associated with an increased risk of NEC. MAB was associated with an increase in infant mortality in one out of three studies. Prolongation of EAB was associated with a significant increase in mortality in three out of eight studies. In the subgroups including extremely preterm infants and studies in which the control group receives only ≤3 days of antibiotics, PEAB was still associated with an increased risk of NEC.

Maternal antibiotic use and its association with NEC development remain controversial. In the three studies included in this meta-analysis, mothers were administered antibiotics in cases of preterm premature rupture of membranes, chorioamnionitis, as GBS prophylaxis, prior to a cesarean section, and for suspected intrauterine infections. We found a negative association between NEC incidence and maternal antenatal antibiotics use. In the large ORACLE-II trial, this effect was not confirmed, and a nonsignificant association was seen between the administration of antibiotics to mothers and the subsequent development of NEC in their infants (32). In addition, a retrospective case–control study also finds a 20% increase in NEC incidence in mothers receiving antenatal antibiotics (29). Potential reasons for these contradicting results are the inclusion of infants born after a gestational age of 32 weeks in the ORACLE-II trial as well as the case–control study by Weintraub et al. A second difference between these studies and the findings of this meta-analysis is the timing of the antibiotics. In these two studies, the mothers received at least a full course of antibiotics, or all antibiotics received throughout the full pregnancy were counted. On the contrary, in this meta-analysis, the largest contributor by Boo and Cheah and the study by Reed et al. only evaluate antibiotics given shortly before giving birth. This included intrapartum antibiotics, and infants in the control group were not exposed to a single dose of antibiotics to prevent wound infection when a cesarean section is performed. The study by Mercer et al. does not specify if mothers in the control group received such a single dose of antibiotics. The subgroup analysis including only the studies by Boo and Cheah and Reed et al. shows no association of MAB with NEC. The effect of antibiotics given shortly before birth can be overshadowed by confounding factors, such as being born by cesarean section, feeding methods, or probiotic supplementation (58). In the two studies reporting on this, the use of cesarean sections was higher in the control group. Finally, the effect of maternal antibiotic use is difficult to discern when neonatal antibiotics are initiated immediately postpartum (57). Potentially, antibiotics given shortly before birth or intrapartum will only result in transient changes in the microbiome, that are not associated with NEC in infants born VLBW or <32 weeks (15, 59, 60).

For reasons of potential inclusion bias, we did not compare any antibiotic use with no antibiotic use in preterm-born infants in this meta-analysis. Two studies included in our meta-analysis do investigate this comparison, showing a higher incidence of NEC in groups receiving empiric antibiotics compared with groups receiving no antibiotics after birth (51, 55). However, several older RCTs on this topic show no significant differences between groups (39, 41, 42). The findings of this meta-analysis are in line with a recent review that compares prolonged EAB with nonprolonged or no antibiotic use in preterm-born infants (61). They report a pooled OR of 2.35 (95% CI: 1.54–3.57) for unadjusted analyses, with a similar effect in adjusted analyses. In addition, they report an increased risk of NEC using gentamicin and meropenem. This meta-analysis did not include the type of antibiotics but did report a large variety in the antibiotics that were used.

A second review on antibiotics and neonatal outcomes also confirms that prolongation of antibiotics in uninfected preterm infants was associated with an increased risk of developing NEC (62).

The main strength of this study is the full exposure of neonate to antibiotics , both *in utero* through maternal antibiotics use and after birth. In addition, the results of all subgroup analyses also indicate that PEAB is associated with an increased risk of NEC. This increases our confidence in the results of this meta-analysis. Only one study evaluated the effect of prolongation of antibiotics after 48 h and found no differences in NEC incidence between both groups (17).

We do acknowledge several limitations. We could only include one RCT in the total of 15 studies that we evaluated. Furthermore, substantial heterogeneity was present among cohort and case–control studies comparing PEAB with EAB. This might be due to variations in the patient population or the duration of empirical antibiotic exposure in the control group. Further analyses identified the study of Al-Mouqdad et al. (48) as a significant contributor to this heterogeneity and the outlier in the funnel plot. In this study, the prevalence of infants with NEC reached 95.1% in the PEAB group, much higher than all other included studies. In addition, 91.2% of those receiving PEAB had a positive blood culture result. After excluding this study, PEAB was still associated with an increased risk of NEC. In this meta-analysis, we did not correct for early onset sepsis with positive blood cultures. Antibiotic therapy is crucial for treating culture-proven sepsis, and thus warrants PEAB. Finally, we included only the data unadjusted for potential confounders in our meta-analysis, and therefore did not assess the effect of these potential confounders. It is reasonable to believe adjusting for confounders would impact the outcome of this meta-analysis, since the observational cohort studies included did not report significant odds ratios after correcting for confounders. However, a recent systematic review focusing on prolonging antibiotics for more than five days found a similar NEC risk when comparing adjusted and unadjusted data (61).

Neonates who are exposed to MAB or PEAB as newborns show a reduction in microbiota diversity and unfavorable microbiota alterations, which are known to predispose NEC (8, 11, 15, 18). The findings of this meta-analysis led us to believe that maternal antibiotic use in preterm or VLBW infants are associated with a reduced NEC incidence. It supports the theory that a prolongation of empiric antibiotic treatment after birth is associated with an increased incidence of NEC and decreased infant safety. One study in this meta-analysis reports on both maternal antibiotics as well as prolonged empiric antibiotics, though not all the data could be incorporated in the pooled odds ratios (44). Their results reflect those of this meta-analysis, as they report a protective effect of maternal antibiotics in infants and a negative association of prolonged EAB>5 days with NEC.

Currently, despite a large number of interventions that have been researched in the past years, none have a high certainty of evidence for NEC prevention (63). Therefore, it is of utmost importance to alter our current standard practices to decrease the risk of NEC development. Based on the results of this meta-analysis, we would advise using empiric antibiotics for as short as possible. This is in accordance with recent guidelines on antibiotics for neonatal infections that recommend evaluating the necessity of antibiotics at 36 to 48 h (64, 65). High quality RCTs, including registration of probiotic administration, breast milk, formula, or donor milk use, and an antibiotic stewardship program, are necessary to confirm our findings.

## Data Availability

The original contributions presented in the study are included in the article/Supplementary Material, further inquiries can be directed to the corresponding author.
