# Supplementary material for: Fetal–neonatal exposure to antibiotics and NEC development: A systematic review and meta-analysis
Source: Front Pediatr. 2023 Jan 16;10:1102884. doi: 10.3389/fped.2022.1102884 (PMC9885048; doi:10.3389/fped.2022.1102884)
Supplement: Supplementary file 2 [file Table2.docx]

**Supplemental File 2**

**MEDLINE search strategy**

("Infant, Premature"[Mesh] OR "Infant, Premature, Diseases"[Mesh] OR prematur*[tiab] OR preterm[tiab] OR "Infant, Very Low Birth Weight"[Mesh] OR "Infant, Extremely Low Birth Weight"[Mesh] OR "Infant, Low Birth Weight"[Mesh] OR low birth weight[tiab] OR low birthweight[tiab] OR VLBW[tiab] OR ELBW[tiab]) AND ("Anti-Bacterial Agents"[Mesh] OR "Anti-Bacterial Agents" [Pharmacological Action] OR antibiotic*[tiab] OR antibacterial*[tiab] OR anti bacterial*[tiab]) AND ((antenatal*[tiab] OR prenatal*[tiab] OR matern*[tiab] OR perinatal*[tiab] OR prepartum[tiab] OR intrapartum[tiab]) OR (empiric*[tiab])) AND ("Enterocolitis, Necrotizing"[Mesh] OR necrotizing enterocolitis[tiab] OR necrotising enterocolitis[tiab]).

**EMBASE**

('antibiotic agent'/exp OR antibiotic*:ab,ti OR 'anti bacterial':ab,ti OR antibacterial:ab,ti) AND ('enterocolitis'/exp OR enterocolitis:ab,ti) AND (('prematurity'/exp OR prematur*:ab,ti OR 'pre matur*':ab,ti OR preterm*:ab,ti OR 'pre term*':ab,ti) OR ('very low birth weight'/exp OR vlbw:ab,ti OR elbw:ab,ti OR 'low birth weight':ab,ti)) AND (('prenatal'/exp OR prenatal:ab,ti OR 'pre natal':ab,ti OR antenatal:ab,ti OR 'ante natal':ab,ti) OR ('perinatal period'/exp OR perinatal:ab,ti OR prepartum:ab,ti OR intrapartum:ab,ti OR maternal:ab,ti) OR empiric*:ab,ti)

**Cochrane Reviews Library**

([mh "Infant, Premature"] OR [mh "infant, premature, diseases"] OR premature*:ti OR preterm*:ti OR [mh "Infant, Low Birth Weight"] OR "low birth weight":ti OR "low birthweight":ti OR "VLBW":ti OR "ELBW":ti)

AND_([mh "Anti-Bacterial Agents"] OR antibiotic*:ti OR antibacterial*:ti OR "anti bacterial*":ti)

AND_(antenatal*:ti OR prenatal*:ti OR matern*:ti OR perinatal*:ti OR prepartum*:ti OR intrapartum*:ti OR empiric*:ti)

AND_([mh "Enterocolitis, Necrotizing"] OR "necrotizing enterocolitis":ti OR "necrotising enterocolitis":ti)
